# Genome-wide analysis of the TIFY family and function of *CaTIFY7* and *CaTIFY10b* under cold stress in pepper (*Capsicum annuum* L.)

**DOI:** 10.3389/fpls.2023.1308721

**Published:** 2023-11-22

**Authors:** Xiaodi Wang, Ning Li, Tianxiang Zan, Kai Xu, Shenghua Gao, Yanxu Yin, Minghua Yao, Fei Wang

**Affiliations:** ^1^ Hubei Key Laboratory of Vegetable Germplasm Innovation and Genetic Improvement, Cash Crops Research Institute, Hubei Academy of Agricultural Sciences, Wuhan, China; ^2^ College of Horticulture and Gardening, Yangtze University, Jingzhou, China; ^3^ Hubei Hongshan Laboratory, Wuhan, Hubei, China

**Keywords:** pepper (*Capsicum annuum* L.), CaTIFY, genome-wide analysis, cold stress, gene expression

## Abstract

TIFY [TIF(F/Y)XG] proteins are a plant particular transcription factor family that regulates plant stress responses. Therefore, to fill this gap, we investigated *CaTIFY* genes in pepper. Gene structure and conserved motifs of the pepper TIFY gene family were systematically analyzed using sequence alignment analysis, *Cis*-acting element analysis, transcriptomic data, and RT-qPCR analysis, and their expression patterns were further analyzed using Virus-Induced Gene Silencing (VIGS) and cold stress reactive oxygen species (ROS) response. We identified 16 *CaTIFY* genes in pepper, which were dispersed among seven subgroups (JAZI, JAZII, JAZIII, PPD, TIFY, and ZIM/ZML). Several *CaTIFY* members had stress-related harmonic-responsive elements, and four (*CaTIFY7*, *CaTIFY10b*, *CaTIFY1b*, and *CaTIFY6b*) had low-temperature-responsive elements. Transcriptomic data and RT-qPCR analysis revealed that the TIFY genes in pepper displayed different expression patterns in the roots, stems, leaves, flower fruits, and seeds. In particular, *CaTIFY7* was highly expressed in young leaves, and *CaTIFY10b* was highly expressed in roots. *CaTIFY*s participated in the regulation of several different abiotic stresses and *CaTIFY7* and *CaTIFY10b* were significantly induced by cold stress. Additionally, Virus-Induced Gene Silencing (targeting *CaTIFY7* and *CaTIFY10b*) resulted in plants that were sensitive to cold stress. Conversely, overexpression of *CaTIFY7* and *CaTIFY10b* enhanced plant cold tolerance by promoting the expression of genes related to cold stress and the ROS response. CaTIFY7 and CaTIFY10b interacted with themselves and CaTIFY7 also interacted with CaTIFY10b in the yeast two-hybrid (Y2H) system. Our data provide a basis for further analysis of the role of pepper TIFY genes in cold-stress responses in the future.

## Introduction

The *TIFY* gene family encodes a transcription factor unique to plants characterized by a highly conserved TIFY domain, which plays an important regulatory role in plant growth, development, and stress tolerance. The TIFY domain is composed of 36 amino acids and a TIF[F/Y] XG core region (Vanholme et al., 2007). Many studies have shown that the TIFY family of proteins can be divided into four subfamilies: TIFY, JAZ, PPD, and ZML (Bai et al., 2011). The first TIFY gene was identified in *Arabidopsis thaliana* (Nishii et al., 2000). Subsequently, the TIFY gene family was identified in rice (Ye et al., 2009), maize (Heidari et al., 2021), cassava (Zheng et al., 2022), tomato (Heidari et al., 2021), and other species (Huang et al., 2016; Sirhindi et al., 2016; Xia et al., 2017; He et al., 2020; Du et al., 2022).

The TIFY protein is a key regulator of the jasmonic acid signaling pathway, participates in defense and stress responses during plant development, and plays a major role in responses to biological and abiotic stresses (Thireault et al., 2015; Ebel et al., 2018). There are few reports on the response of the TIFY family to biotic stress, mainly focusing on the abiotic stress response of the TIFY family. Multiple TIFY genes have been shown to respond to drought and salt stress in arabidopsis, apples, grapes, rice, soybeans, and corn (Ye et al., 2009; Zhang et al., 2012; Li et al., 2015; Zhou et al., 2015; Meng et al., 2019; Zhao et al., 2020). In rice, the overexpression of *OsTIFY11a* significantly improves its tolerance to salt and dehydration stress (Ye et al., 2009). In addition, OsJAZ1 interacts with OsbHLH148, a transcription regulator in the jasmonic acid signaling pathway, resulting in enhanced drought tolerance in rice (Seo et al., 2011). Among solanaceous plants, only the TIFY genes of tomato have been identified, and transcriptome data show that most *SlJAZ* genes respond to salt, osmotic stress, or JA-ABA treatments (Chini et al., 2017).

With the rapid development of transcriptome sequencing, an increasing number of TIFY genes have been found to respond to low-temperature stress (Ye et al., 2009; Zhang et al., 2012; Zhang et al., 2015; Huang et al., 2016; He et al., 2020). Most *BdTIFY* genes are responsive to one or more abiotic stressors, including drought, salinity, low temperature, and heat (Zhang et al., 2015). In grapes, *VvJAZ4*, *5*, *9*, *11*, and *VvZML1* respond to cold stress and are regulated by drought or salinity (Zhang et al., 2012). In *Brassica napus*, recent research has found that *TIFY11b*, *TIFY10a*, *TIFY3*, *TIFY5a/b*, and *TIFY6* are induced by cold acclimation and overexpression of *BnaJAZ7⁃A3/BnaJAZ7⁃C3* enhances cold injury stress (He et al., 2020). In rice, some *OsTIFY* genes are induced by cold but not induced or even repressed by ABA, which suggests *OsTIFY* participated in the cold-stress response via ABA-independent signaling pathways (Ye et al., 2009). Studies on Arabidopsis have found that the interaction between JAZ1/JAZ4 and C-repeat binding factor (CBF) inducers inhibits the function of transcriptional ICE1, thereby weakening the expression of CBFs (Huang et al., 2012; Hu et al., 2013). Furthermore, the overexpression of *JAZ1* or *JAZ4* inhibits the response of Arabidopsis to freezing stress (Hu et al., 2013). However, few studies have revealed the function of the TIFY protein in plant low-temperature responses.

Pepper (*Capsicum annum* L.) is a versatile vegetable belonging to the Solanaceae family and is an economically important crop worldwide. The average yield loss caused by low temperatures is enormous (Chinnusamy et al., 2007; Legris et al., 2017). Although there have been many advances in understanding the cold resistance mechanisms of crops, there are still few reports on the low-temperature tolerance of pepper. Transcription factors also play an important role in the response of pepper to low-temperature stress; however, only a small number of transcription factor families, including MADS, NAC, and bHLH, have been shown to participate in the low-temperature regulation process in pepper (Guo et al., 2015; Chen et al., 2019; Hou et al., 2020; Zhang et al., 2020; Wang et al., 2022; Zhang et al., 2023). So far, the TIFY protein in pepper has not been identified, and its role in the cold-stress response has not been studied. Therefore, to fill this gap, we investigated *CaTIFY* genes in pepper.

In this study, we identified 16 genes encoding TIFY proteins in the pepper genome. Chromosomal position, phylogenetics, gene structure, conserved domains, and promoter cis-element analyses were carried out to provide genome-wide identification and investigation of CaTIFY genes. Furthermore, the expression of all CaTIFY genes was investigated in various tissues and organs at different developmental stages and under various abiotic stresses, using published transcriptome data. We also examined their expression profiles after cold treatments and in different tissues by RT-qPCR assays and found *CaTIFY7* and *CaTIFY10b* were induced by cold stress in leaves significantly. Gene function analysis of *CaTIFY7* and *CaTIFY10b* showed that they positively regulated cold-stress tolerance in pepper plants. Further analysis demonstrated that the downregulation or overexpression of *CaTIFY7* and *CaTIFY10b* influenced the expression levels of cold-stress and reactive oxygen species (ROS)-related genes. Further, the Y2H assay proved CaTIFY7 and CaTIFY10b interacted with themselves and CaTIFY7 also interacted with CaTIFY10b. By performing this study, we have laid a foundation for further research on the function and molecular mechanism of the TIFY gene family in the pepper stress response and provide a basis for improving plant stress resistance in the future.

## Materials and methods

### Identification of CaTIFY gene family

TIFY domain (PF06200) hidden Markov file was downloaded from Pfam database (http://pfam.xfam.org/). HMMER software was used to screen for possible TIFY proteins in the pepper genome and candidate members were submitted to Pfam and SMART (https://smart.embl.de/) online programs to verify the composition of conservative structural domains for further screening.

### Gene structure analysis

The CDS and genome sequence of the pepper TIFY gene family were submitted to the GSDS (http://gsds.gao-lab.org/) online website for visual analysis of the intron–exon substructure of the TIFY gene family.

### Functional structural domain analysis

TBtools was used to extract the TIFY protein sequence of pepper and MEGA software was used to perform multiple sequence alignments on the TIFY protein sequence of pepper. The functional domain of the TIFY protein in chili pepper was analyzed based on conserved domain sequence characteristics.

### Phylogenetic tree analysis

First, we used MEGA5 software to conduct multiple sequence alignments of Arabidopsis, rice, tomato, and identified pepper TIFY protein sequences. Alignment results were used to build a phylogenetic tree based on the maximum parsimony method, and the bootstrap value was set to 1000. Next, the online program Itol (https://itol.embl.de/) was used to further refine the evolutionary tree.

### Chromosome localization analysis

We obtained the genome sequences and GFF files of peppers from the PepperHub database (http://122.205.95.132/index.php) (Liu et al., 2017). TBtools software was used to extract the chromosome length and location information of TIFY genes in the pepper genome from the GFF files and to perform visual analysis.

### Conserved motifs analysis of CaTIFY proteins by MEME

Conserved motifs in CaTIFYs were predicted using MEME-Suite (https://meme-suite.org/meme/tools/meme) and visualized using TBtools. Specific parameters were set as follows: ‘Any number of repetitions’ was selected as the site distribution, and up to ten motifs were retrieved.

### Promoter *cis*-acting elements analysis

First, 2000 bp upstream DNA sequences of the pepper TIFY gene CDS were extracted from the pepper genome sequence using the TBtools software. We then uploaded these sequences to the PlantCARE database (http://bioinformatics.psb.ugent.be/webtools/plantcare/html/) to obtain *cis*-acting element information on the promoter of CaTIFY genes in pepper. Subsequently, the *cis*-acting elements related to stress and hormones were classified. TB tools software was used to visualize the analysis of CaTIFY gene promoters in pepper.

### Protein interaction network prediction

The identified BvTIFY protein sequence was uploaded to the STRING database (https://cn.string-db.org/) for prediction of the biological functions of homologous CaTIFY and other proteins and their potential interactions.

### Expression patterns of GmTIFY genes under abiotic stresses

RNA-seq data of CaTIFY family members in different tissues and organs (roots, shoots, young leaves, mature leaves, old leaves, flowers, fruits, and seeds) under several abiotic stresses (salt, drought, H_2_O_2_, heat, and cold) were obtained from the PepperHub Database. HEMI software was used to visualize the expression levels of CaTIFYs.

### Plant materials and cold-stress treatment

Peppers (*Capsicum annuum* L.) were cultivated under a 25°C temperature and 16/8-h day/night photoperiod. Pepper plants of uniform size were selected and exposed to cold stress (4°C) at the 6-8 true leaf stage in growth chambers. Leaves and roots were sampled at 1, 3, 6, 12, and 24 h after cold treatment.

### Real-Time Fluorescence Quantitative PCR (RT-qPCR)

TRIzol and chloroform reagents were used to extract total RNA from pepper leaves and roots. A HiScript II 1st Strand cDNA Synthesis Kit (+ gDNA wiper) (Vazyme, Nanjing, China) was used for reverse transcription. ChamQ Universal SYBR^®^ qPCR Master Mix (Vazyme) and CFX384 Real-Time PCR Detection System (Bio-Rad, Hercules, CA, USA) were used to perform RT-qPCR. The quantitative results were analyzed by the 2^−ΔΔCt^ method and the CaUBI3 gene (Wan et al., 2011) was used as the internal control for normalizing gene expression. Each experiment was performed in triplicate. The primers used in the qPCR analysis are listed in **Supplementary Table 1**.

### Virus-Induced Gene Silencing (VIGS) assay in pepper

The silence-specific 312 bp fragment of *CaTIFY7* was selected and inserted into the pTRV2 vector between the KpnI and XhoI sites to form the pTRV2:*CaTIFY7* recombinant plasmid. The pTRV2:*CaTIFY7*, pTRV2, and pTRV1 were individually transformed into Agrobacterium GV3101 cells. Suspensions of pTRV2:*CaTIFY7* and pTRV2 were individually mixed with equal volumes of pTRV1 and injected into the cotyledons of two true-leaf-stage pepper seedlings as described previously (Wang et al., 2013). The same experimental method was used for *CaTIFY10b* and specific 331 bp fragments of *CaTIFY10b* were used to build a carrier.

### Transient expression of *CaTIFY7* and *CaTIFY10b* in pepper leaves

The full-length *CaTIFY7* cDNA (Capana01g003720) and *CaTIFY10b* cDNA (Capana07g000750) were obtained from the Pepper Genome Database. PCR-amplified products of *CaTIFY7* and *CaTIFY10b* were cloned into empty vectors to generate 35S:*CaTIFY7* and 35S:*CaTIFY10b*. The empty vectors 35S:*CaTIFY7* and 35S:*CaTIFY10b* were individually transformed into Agrobacterium GV3101. The suspensions were infiltrated into the leaves of pepper plants at the eight-leaf stage as described previously (Cai et al., 2021). After 48 h, half of the infiltrated plants were moved to 4 °C treatment. All infiltrated leaves were collected for further use 24 h after cold stress.

### Cold tolerance indices in pepper

To assess cold-stress biochemical indices, 3,3’-diaminobenzidine (DAB) and nitro blue tetrazolium staining (Coolaber, Beijing, China) were used to stain the transformed leaves as per the manufacturer’s instructions.

### Yeast two-hybrid (Y2H) assay

To determine the relationship between CaTIFY7 and CaTIFY10b, we performed a Y2H assay using the MatchmakerTM Two-Hybrid System (Clontech, USA). The CDS of CaTIFY7 and CaTIFY10b were amplified with specific primers and cloned into the pGBKT7-BD vector between the *Eco*RI and *Pst*I sites and the pGADT7-AD vector between the *Eco*RI and *Bam*HI sites. The fusion pGBKT7-BD and pGADT7-AD constructs were co-transformed into the Y2H Gold strain and screened for SD/-Leu/-Trp. Then multiple monoclonal clones were mixed and spotted onto SD/-Leu/-Trp/-His/-Ade media at 30°C for 3-5 d. The fusion of pGBKT7-53 and pGADT7-T was used as a positive control.

## Results

### Identification of TIFY members in pepper

**Figure 1:**
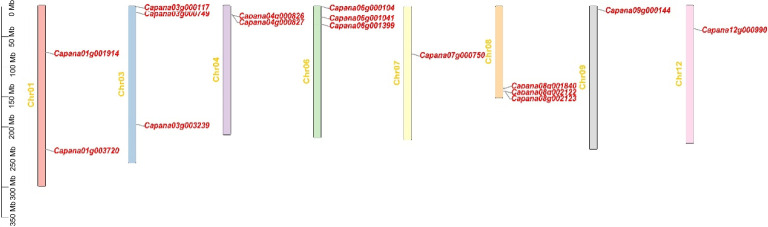
Distribution of the TIFY genes on chromosomes of pepper. The scale on the right is in million bases (Mb).

To identify TIFY members in pepper, the TIFY domain (PF06200) was used to screen for possible TIFY proteins in the pepper genome. 16 *TIFY* genes were identified in the pepper genome and named CaTIFYs, according to their homologous TIFY genes in Arabidopsis (**Figure 1**). The numbers of amino acids in CaTIFYs ranged from 135 to 411 and their molecular weights ranged from 14.61 to 45.7 KDa. The theoretical PIs of the CaTIFYs ranged from 4.65 to 10.68 (**Table 1**). In addition, the subcellular location prediction analysis showed that all CaTIFYs were located in the nucleus.

**Table 1:** CaTIFY gene feature information in pepper.

Gene name	Locus ID	Chromosome	Starting site	Ending site	ORF(aa)	CDS(bp)	MW(kDa)	pI	Subcellular location
CaTIFY9	Capana01g001914	Chr01	78262060	78263252	153	459	17.11	6.69	Nucleus.
CaTIFY7	Capana01g003720	Chr01	239982070	239985708	346	1038	36.56	8.9	Nucleus.
CaTIFY10a	Capana03g000117	Chr03	1604329	1606207	299	897	32.47	9.17	Nucleus.
CaTIFY6a	Capana03g000749	Chr03	10975774	10979194	329	987	33.89	9.52	Nucleus.
CaTIFY4a	Capana03g003239	Chr03	196914896	196921253	411	1233	45.7	8.71	Nucleus.
CaTIFY2	Capana04g000826	Chr04	16098710	16101334	269	807	29.78	7.33	Nucleus.
CaTIFY1a	Capana04g000827	Chr04	16106805	16109264	168	504	18.79	4.65	Nucleus.
CaTIF Y4b	Capana06g000104	Ch06	1376792	1379848	378	1134	42.06	8.84	Nucleus.
CaTIFY6b	Capana06g001041	Chr06	18388538	18393631	300	900	33.56	10.68	Nucleus.
CaTIFY8	Capana06g001399	Chr06	30846359	30852144	383	1149	40.09	9.87	Nucleus.
CaTIFY10b	Capana07g000750	Chr07	79075974	79076928	229	687	25.15	9.58	Nucleus.
CaTIFY1b	Capana08g002122	Chr08	141545179	141560486	340	1020	35.71	6.74	Nucleus.
CaTIFY1c	Capana08g002123	Chr08	141562343	141568832	374	1122	41.07	4.92	Nucleus.
CaTIFY3	Capana08g001840	Chr08	137205501	137206502	135	405	14.61	7.51	Nucleus.
CaTIFY10c	Capana09g000144	Chr09	5548125	5549825	251	753	27.93	10.09	Nucleus.
CaTIFY10d	Capana12g000990	Chr12	39269034	39274278	209	627	23.04	10.36	Nucleus.

### Conserved domain and motif analysis of CaTIFY proteins

**Figure 2:**
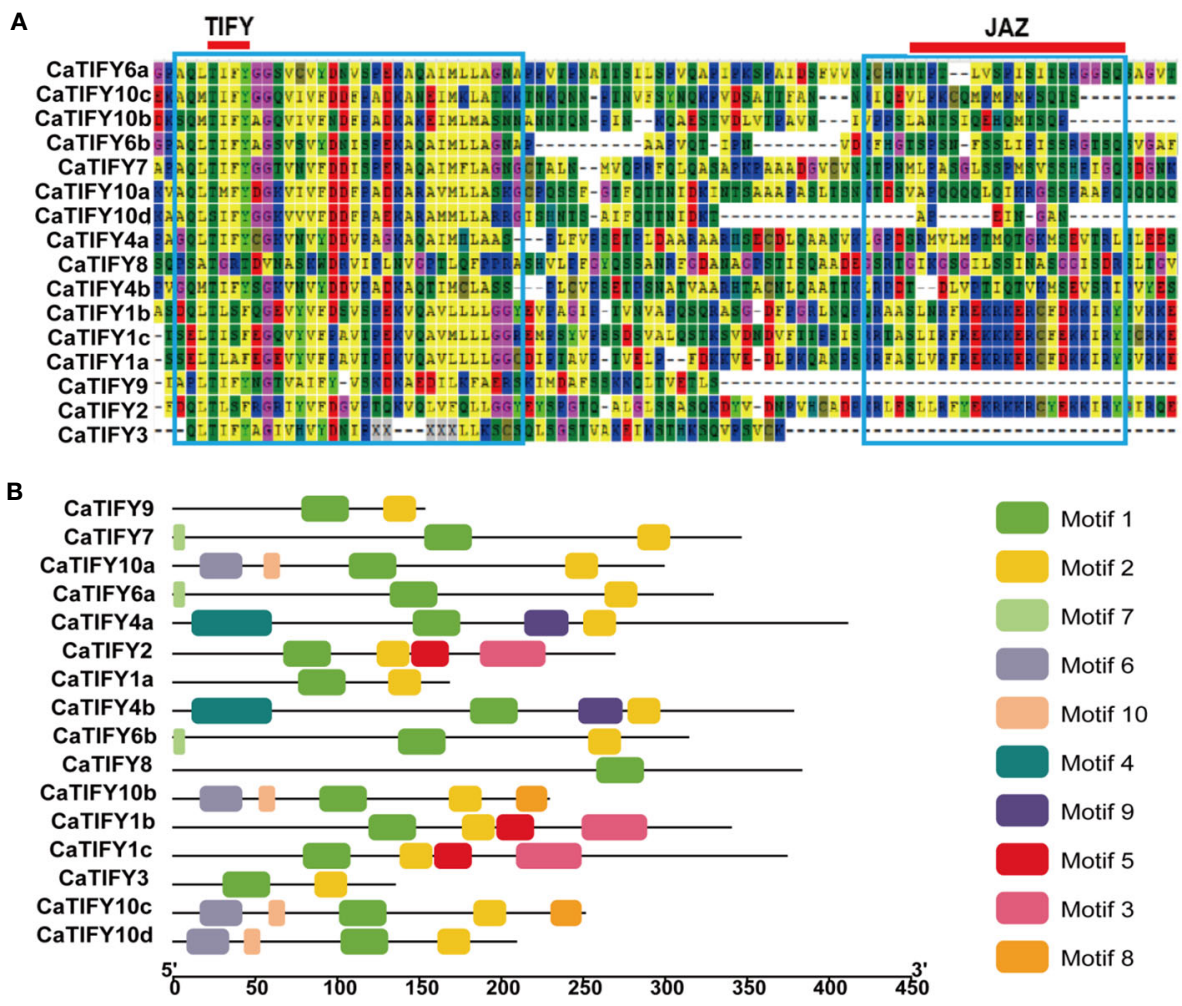
Multiple sequences alignment of CaTIFYs and conserved motif distributions of CaTIFYs proteins in pepper. **(A)** The blue boxes indicate conserved amino acids. The red line indicate the core amino acids of TIFY and Jas domain. **(B)** Distribution of the conserved motifs in pepper TIFY proteins. Ten conserved motifs are marked with different colored boxes. The scale bar indicates 50 aa.

Multiple sequence comparisons revealed that all members of the TIFY gene family contained TIFY and Jaz domains (**Figure 2A**). The TIFY domain of the CaTIFY proteins was more conserved than the Jaz domain. CaTIFY3, CaTIFY9, and CaTIFY10d contained almost no Jaz domains (**Figure 2A**). In addition, conserved motifs were also identified. As shown in **Figure 2B**, a total of 10 motifs were predicted in CaTIFY proteins using the MEME website and TBtools software. The numbers of CaTIFY motifs ranged from one (CaTIFY8) to five (CaTIFY10b/c). There were four motifs in the seven CaTIFY (CaTIFY1b, CaTIFY1c, CaTIFY2, CaTIFY4a, CaTIFY4b, CaTIFY10a, and CaTIFY10d) proteins and their proportions exceeded 30% of the total proteins. Almost all proteins had motif1 and motif2, except for CaTIFY8, which only had motif1. Proteins in the same clade (for example CaTIFY10a, CaTIFY10b, CaTIFY10c, and CaTIFY10d) contained similar conserved motifs. However, CaTIFY1a had only two motifs, unlike CaTIFY1b and CaTIFY1c which had four motifs.

### Phylogenetic relationships and gene structures of *TIFY* genes in pepper

**Figure 3:**
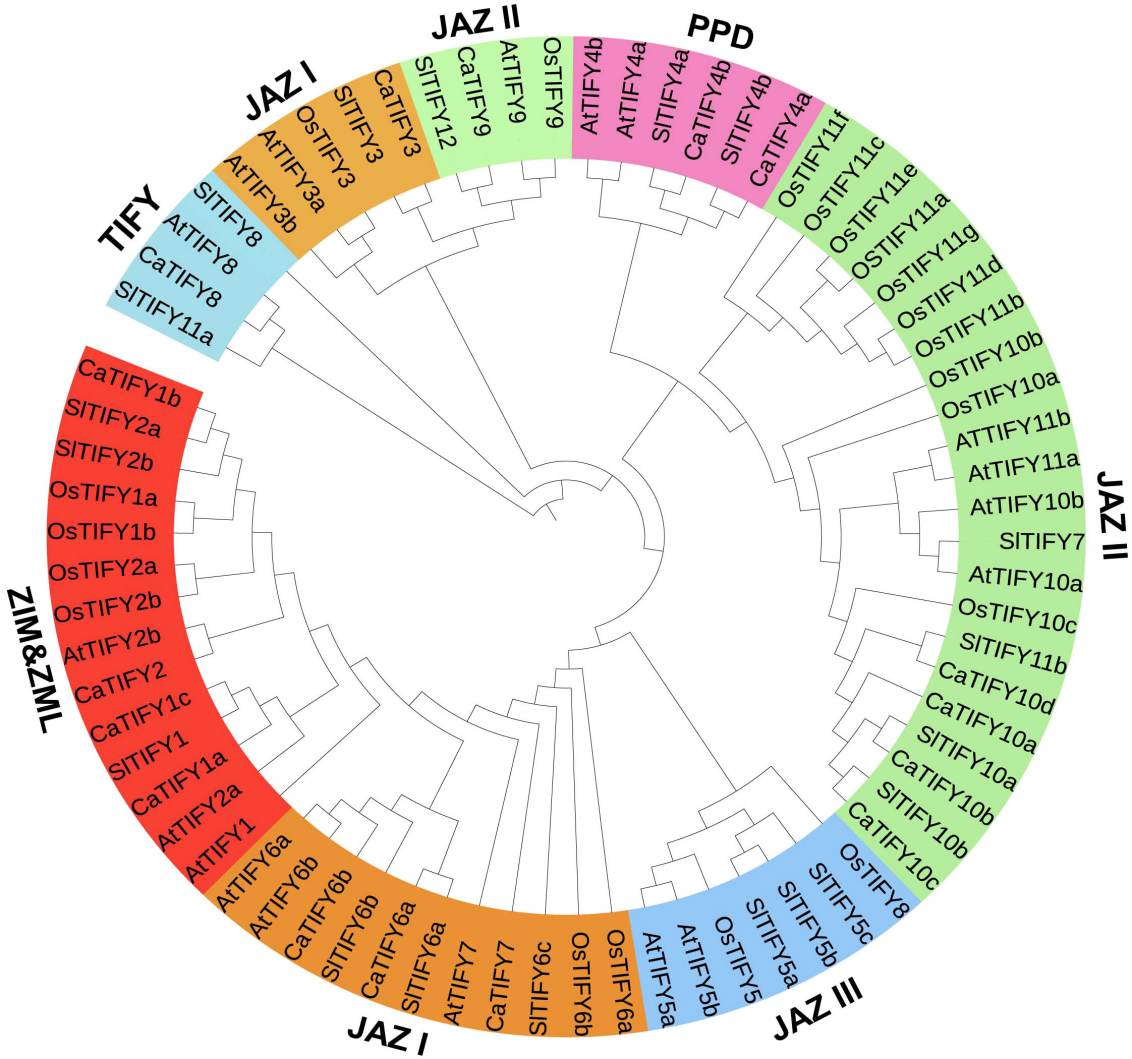
Evolutionary relationship of the TIFY proteins across different plant species. 58 TIFY proteins come from Arabidopsis (18), rice (20), tomato(19), and pepper(16).The resulting 9 groups are classified in four subfamilies, which belong to JAZ, PPD, TIFY and ZIM/ZML.

To investigate the phylogenetic relationships among the CaTIFYs, Pepper CaTIFY, rice OsTIFY, Arabidopsis AtTIFY, and tomato SlTIFY sequences were used to construct a phylogenetic tree (**Figure 3**). Phylogenetic analysis showed that CaTIFY proteins could be divided into 11 groups belonging to the JAZ, PPD, TIFY, and ZIM/ZML groups. In pepper, no TIFY gene belonged to JAZ7/8 (the AtTIFY5 homologous gene). Among the CaTIFYs, TIFY10 had the highest number of homologous genes. Rice has 11 homologous genes of TIFY10/11 and there were four homologous genes in pepper. Notably, although tomatoes and peppers are Solanaceae plants, there are only four TIFY homologous genes in tomatoes.

To understand the structural diversity of TIFY genes, we further analyzed the structure of the *CaTIFY* gene family. The intron–exon substructures of *CaTIFY* genes are visualized in **Supplementary Figure 1**. According to the evolutionary relationships of the CaTIFY protein, there were significant differences in sequence length and structure among genes, indicating that large sequence differences existed among the different *CaTIFY* genes (**Supplementary Figure 1**). The number of CaTIFY introns ranged between 1 and 9, of which CaTIFY1c contained nine introns and CaTIFY9 had only one. All other CaTIFY intron numbers ranged from 2 to 8. There were no strong correlations between phylogeny and exons/introns. Genes with high homology did not have similar gene structures and the length of introns varied greatly, indicating significant gene differentiation in the TIFY family in pepper.

### Cis-acting element analysis of pepper *TIFY* gene family

**Figure 4:**
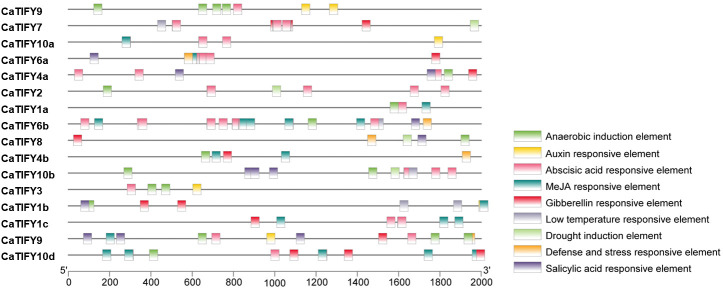
Promoter *cis*-element analysis of *CaTIFY* genes. Nine *cis*-acting elements related to abiotic stress are detected in each species, in which they are represented by distinct colored boxes.

Cis-acting elements can affect and predict gene expression in response to stress or hormones. The 2000 bp region upstream of the start codon of each pepper *TIFY* gene was extracted from the Pepper Genome Database. PlantCARE software was used to analyze the cis-acting elements related to stress or hormones. As shown in **Figure 4**, 81.2% of the members of the TIFY gene family in peppers had anaerobic induction elements. Of the five hormones, 25% of the *CaTIFY* members had auxin-responsive elements and 56.2% of the *CaTIFY* members had ABA-, MeJA-, and gibberellin-responsive elements. Fifty percent of *CaTIFY* members had a salicylic acid-responsive element. In addition, four *CaTIFY* members (*CaTIFY7*, *CaTIFY10b*, *CaTIFY1b* and *CaTIFY6b*) had a low-temperature-responsive element, four *CaTIFY* members (*CaTIFY7*, *CaTIFY2*, *CaTIFY8* and *CaTIFY10b*) had drought induction elements, and four *CaTIFY* members had defense- and stress-responsive elements (**Figure 4**).

### Expression patterns of *CaTIFY* genes in different tissues and under abiotic stresses

To understand the expression patterns of *CaTIFY* genes in different pepper tissues, the relative expression levels of *CaTIFY* family genes in eight tissues (roots, shoots, young leaves, mature leaves, old leaves, flowers, fruits, and seeds) were determined based on published RNA-seq data. As is shown in **Supplementary Figure 3A**, a heat map was generated to illustrate the expression profiles. The relative expression levels of the 16 *CaTIFY* genes differed among the eight tissues (roots, shoots, young leaves, mature leaves, old leaves, flowers, fruits, and seeds). The expression levels of *CaTIFY1c*, *CaTIFY2*, *CaTIFY3*, and *CaTIFY6b* were very low in all tissues. *CaTIFY7* was highly expressed in young leaves, mature leaves, old leaves, flowers, and fruits but had very low transcription levels in roots, shoots, and seeds (**Supplementary Figure 3A**). Significantly, the expression level of *CaTIFY10b* in the roots was the highest among all *CaTIFY* genes, but its expression level in other tissues was relatively low, especially in the leaves and seeds. Additionally, CaTIFY10c showed a tissue-specific expression pattern similar to that of *CaTIFY10b*. However, its overall expression level was lower than that of *CaTIFY10b* (**Supplementary Figure 3A**). Interestingly, although *CaTIFY10d* is homologous to *CaTIFY10a/b/c*, its tissue-specific expression patterns were completely different. *CaTIFY10d* was hardly expressed in roots and seeds but had relatively high expression levels in flowers. Subsequently, we validated the tissue expression patterns of these CaTIFY genes in pepper leaves using RT-qPCR (**Supplementary Figures 2A–P**). Consistent with the transcriptome data, *CaTIFY7* was highly expressed in young leaves and flowers and *CaTIFY10b* was highly expressed in roots (**Supplementary Figures 2J, N**). Overall, most *CaTIFY* genes had the highest expression levels in leaves. The exceptions were *CaTIFY1b* and *CaTIFY6a*, which showed the highest expression levels in roots, and *CaTIFY6b*, which showed the highest expression level in flowers (**Supplementary Figure 2**).

To characterize the abiotic-responsive *CaTIFY* genes, the Fragments Per Kilobase of exon model per million mapped fragments (FPKM) values of each gene in leaves and roots treated with NaCl, mannitol, H_2_O_2_, heat, or cold were determined (**Supplementary Figures 3B–F**). Under salt stress, the transcript levels of *TIFY4a*, *TIFY6b*, and *TIFY8* in leaves were significantly repressed, whereas the expression levels of TIFY6b and TIFY8 in the roots were induced by NaCl treatment. *TIFY10b* and *TIFY10c* were strongly induced by salt stress, especially in roots (**Supplementary Figure 3B**). Mannitol treatment induces drought stress responses in plants. As shown in **Supplementary Figure 3C**, *TIFY7*, *TIFY10b*, and *TIFY10c* were induced by drought stress in the leaves and roots of pepper. *TIFY6a* and *TIFY6b* were strongly induced by drought stress in roots, whereas *TIFY6b* was strongly repressed by drought stress in leaves. H_2_O_2_ is one of the most abundant reactive oxygen species (ROS) in cells, and its accumulation causes oxidative damage to cells. It is a key signaling molecule involved in plant growth, development, and stress resistance. In the leaves, *TIFY6a*, *TIFY7*, *TIFY10a*, *TIFY10b*, and *TIFY10c* were induced by H_2_O_2_ whereas *TIFY6b* was reduced by H_2_O_2_. *TIFY6a*, *TIFY6b*, *TIFY7*, *TIFY8*, *TIFY10a*, *TIFY10b* and *TIFY10c* were induced by H_2_O_2_ in roots while the expression level of *TIFY1a* was reduced by H_2_O_2_ in roots (**Supplementary Figure 3D**).

Temperature is an important factor that restricts plant growth, and either too high or too low a temperature can cause stress on plant growth. Under high-temperature stress, *TIFY6a* and *TIFY7* were strongly repressed, whereas *TIFY1a* and *TIFY10b* were induced by heat stress (**Supplementary Figure 3E**). Additionally, *TIFY6a*, *TIFY6b*, *TIFY10b*, and *TIFY10c* were induced by heat stress (**Supplementary Figure 3E**). As expected, cold stress also affected the expression of some *TIFY* genes in pepper plants. As shown in **Supplementary Figure 3F**, *TIFY10a*, *TIFY10b*, and *TIFY10c* were strongly induced by cold stress, whereas they were not induced by chilling in either roots or leaves. Surprisingly, *TIFY6a* and *TIFY6b* were strongly induced by cold stress in the roots but were repressed by cold stress in the leaves. The expression levels of *TIFY1a* in the roots and leaves reduced under cold treatment. The responses of the other genes to cold stress were not significant.

### RT-qPCR analysis of expression patterns of *CaTIFY* genes under cold stress

**Figure 5:**
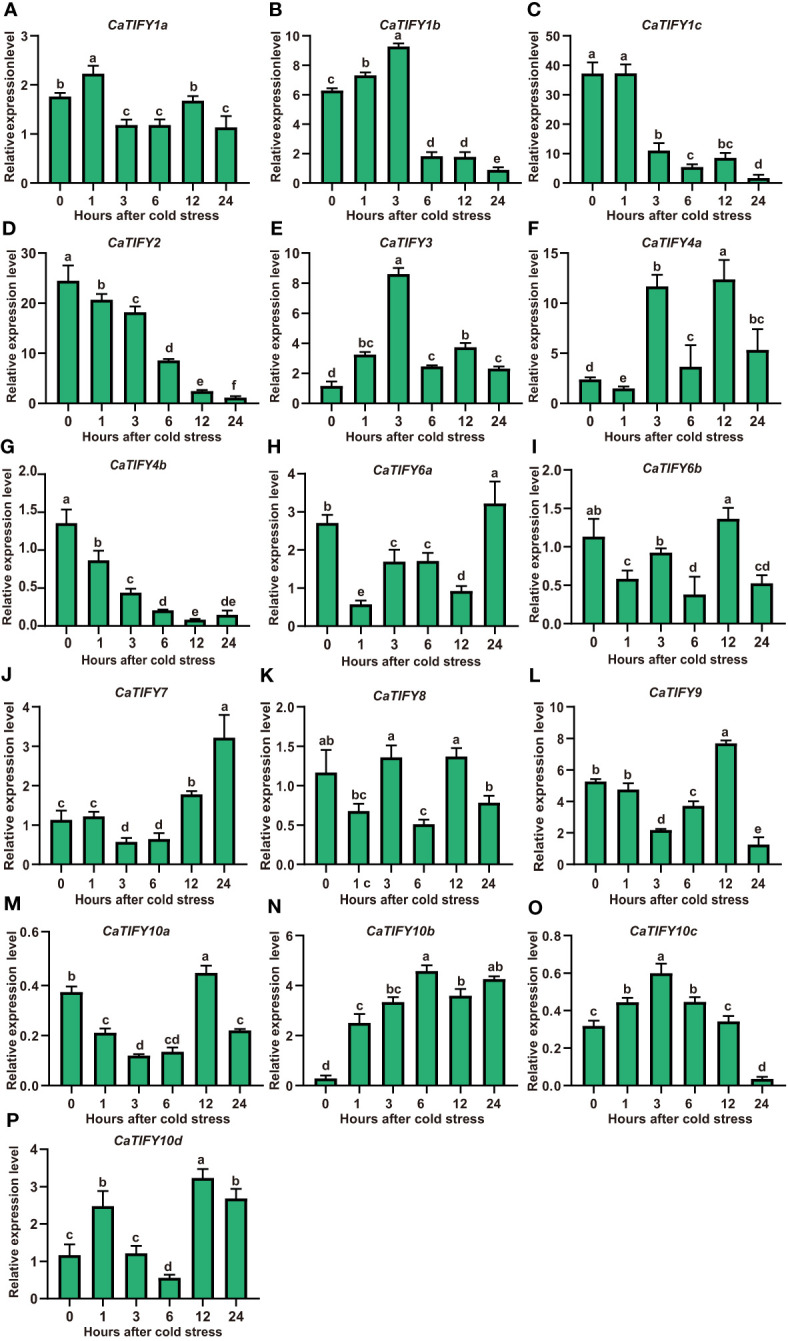
RT-qPCR analysis of expression patterns of *CaTIFY* genes in pepper leaf under cold stress. **(A–P)** Expression levels of sixteen *CaTIFY* genes were measured using RT-qPCR at different times under 4 °C cold stress. RT-qPCR data were normalized using *CaUBI3* as the reference gene and were displayed relative to 0 h. Different letters indicate significant differences between samples according to the Student-Newman-Keuls test (*P <*0.05).

To examine the changes in *CaTIFY* gene expression of pepper in response to cold stress, the pepper seedlings were subjected to 4 °C treatment. We determined the abundance of transcripts of sixteen *CaTIFY* genes under cold stress using RT-qPCR to confirm the results of the RNA-seq data. As shown in **Figure 5**, *CaTIFY7* and *CaTIFY10b* were significantly induced by cold stress in leaves, whereas *CaTIFY1b*, *CaTIFY1c*, *CaTIFY2*, and *CaTIFY4b* were significantly reduced by cold treatment (**Figures 5B–D, G, J, N**). Additionally, the expression of *CaTIFY3*, *CaTIFY6a* and *CaTIFY10d* tended to be induced after cold stress treatment (**Figures 5E, H, P**). However, the constitutive expression levels of *CaTIFY3*, *CaTIFY6a* and *CaTIFY10d* were much lower than *CaTIFY7* and *CaTIFY10b* in leaf and under low-temperature treatment as shown in published transcriptome data (**Supplementary Figures 3A, F**). In summary, all of the results identified *CaTIFY7* and *CaTIFY10b* as candidates for further studies on the potential important roles of *CaTIFY* genes in enhancing low-temperature stress tolerance in pepper leaves.

### *CaTIFY7* enhanced the cold-stress tolerance in pepper

**Figure 6:**
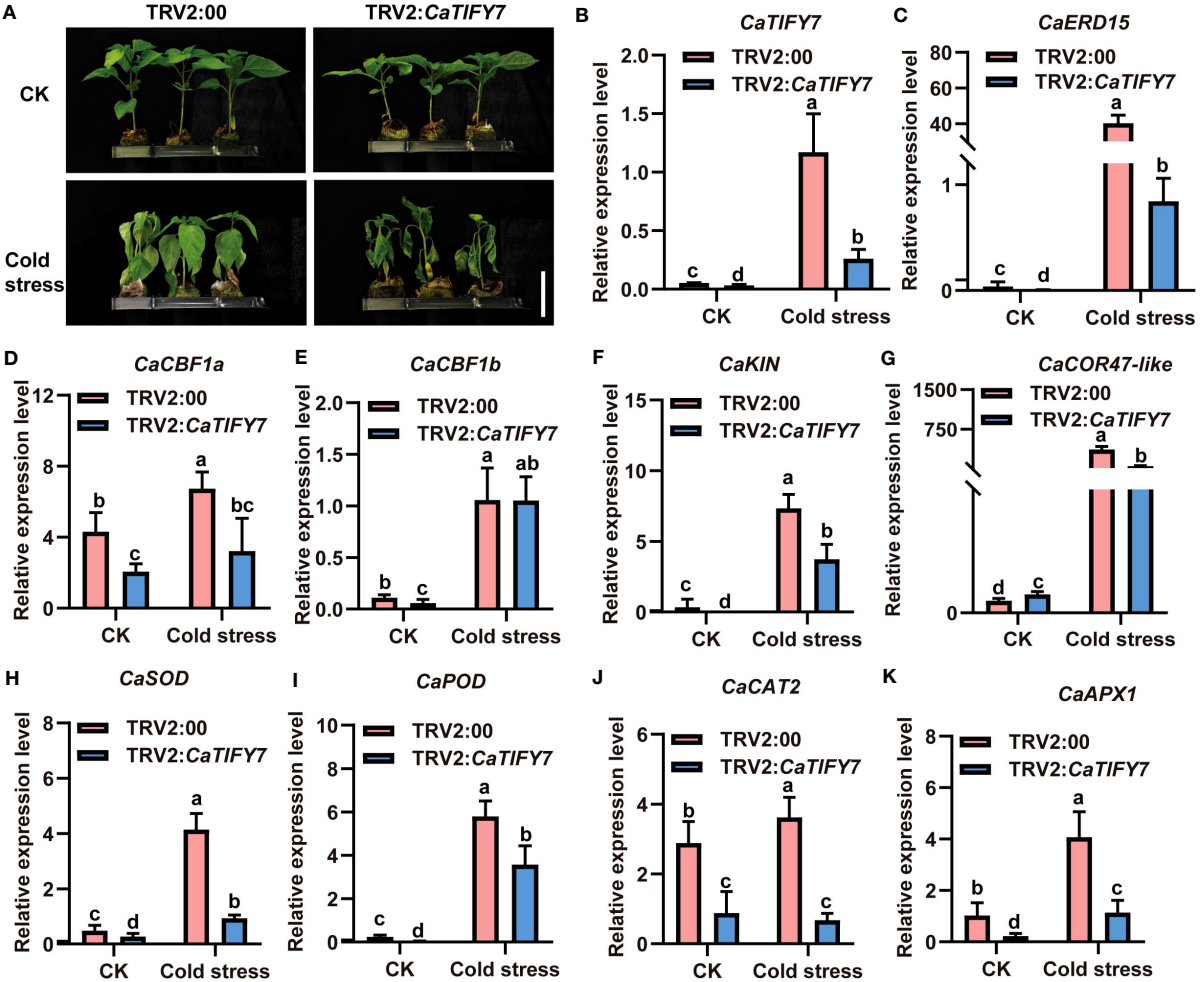
Knock down of *CaTIFY7* in pepper weaken plant tolerance significantly under cold stress. **(A)** The phenotype of pepper plants with reduced expression *CaTIFY7* under 4 °C cold stress for 48 (h) Bar=4 cm. **(B)** The silencing efficiency of *CaTIFY7* under normal or cold stress. **(C-K)** The expression level of *CaERD15*, *CaCBF1a*, *CaCBF1b*, *CaKIN*, *CaCOR47-like*, *CaSOD*, *CaPOD*, *CaCAT2* and *CaAPX1* were analyzed in transgenic pepper plants with TRV2:*CaTIFY7* under normal or cold stress. *CaUBI3* were used as the reference gene. All the values are the averages ± SD from three independent experiments. Different letters indicate significant differences between samples according to the Student-Newman-Keuls test (*P <*0.05).

**Figure 7:**
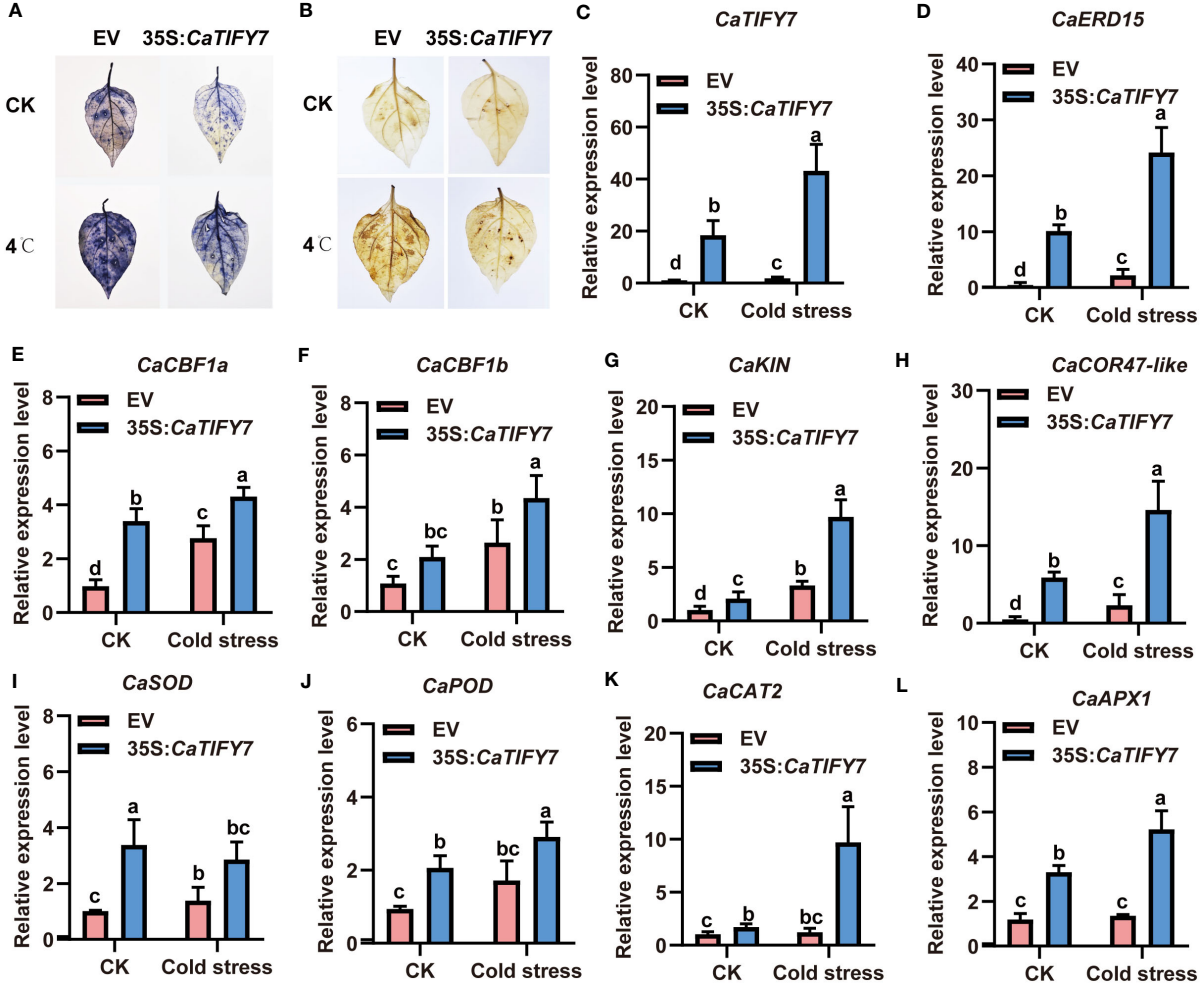
Overexpression of *CaTIFY7* enhances cold stress tolerant in pepper. **(A)** O_2_^-^ detecting with NBT after cold stress. **(B)** H_2_O_2_ staining with DAB under normal or cold stress. **(C)** The expression level of *CaTIFY7* were analyzed in transgenic pepper plants having 35S:*CaTIFY7* under normal or cold stress. **(D-L)** The transcription level of *CaERD15*, *CaCBF1a*, *CaCBF1b*, *CaKIN*, *CaCOR47-like*, *CaSOD*, *CaPOD*, *CaCAT2* and *CaAPX1* were detected in transgenic pepper plants with 35S:*CaTIFY7* after cold stress treatment. The gene expression levels were normalized to those of *CaUBI3*. The relative transcript level was determined and normalized using the reference level and averaged over the three technical replicates. All the values are the averages ± SD from three independent experiments. Different letters indicate significant differences between samples according to the Student-Newman-Keuls test (*P <*0.05).

To confirm the role of *CaTIFY7* in pepper under cold stress, Virus-Induced Gene Silencing (VIGS) was used to knock down the gene. Silencing *CaTIFY7* significantly increased the sensitivity of pepper to cold stress. As shown in **Figure 6A**, pepper plants transformed with TRV2:00 and TRV2:*CaTIFY7-*silenced both showed normal, undifferentiated growth under normal conditions. However, the growth of pepper plants transformed with an empty vector was significantly better than those with *CaTIFY7* under 4 °C cold stress treatment. RT-qPCR analysis revealed that the *CaTIFY7* silencing efficiency was high and showed a 68.6% or 80.6% reduction compared to the control group under normal or cold stress, respectively (**Figure 6B**). *CaTIFY7* knockdown led to significant downregulation of cold-induced genes (*CaERD15*, *CaCBF1a*, *CaCBF1b*, *CaCOR47-like*, and *CaKIN*) and ROS-related genes (*CaPOD*, *CaSOD*, *CaCAT2*, and *CaAPX1*) under cold stress (**Figures 6C–K**). *CaTIFY7* overexpression markedly increased the reactive oxygen species content and expression levels of cold-induced genes and ROS-related genes under cold stress (**Figures 7A–L**). Preliminary results indicated that *CaTIFY7* plays a positive role in regulating pepper tolerance to cold stress.

### *CaTIFY10b* also promoted pepper tolerance to cold stress

**Figure 8:**
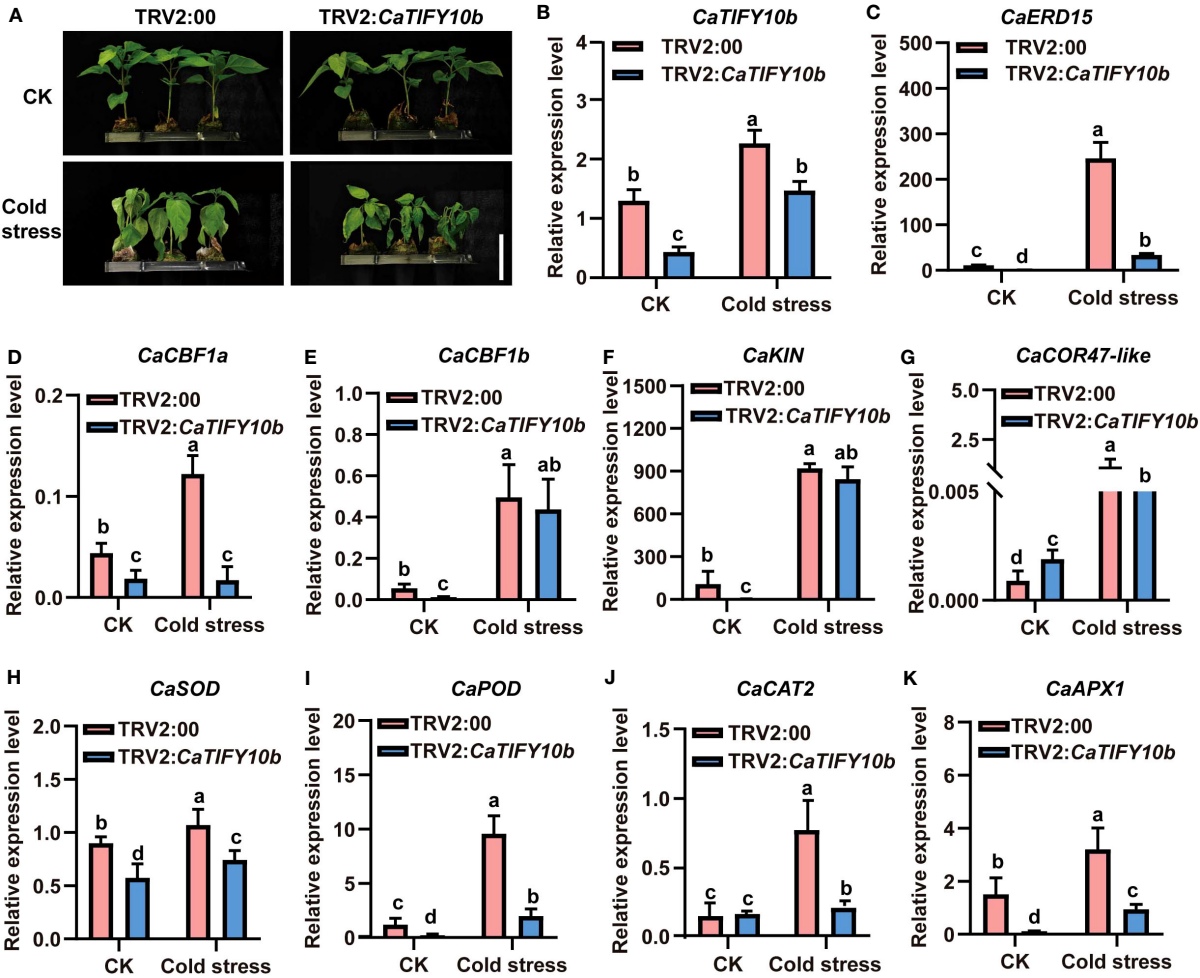
Knock down of *CaTIFY10b* in pepper seedlings improves sensitiveness under cold stress. **(A)** The phenotype of pepper plants with suppression of *CaTIFY10b* under cold stress for 48 **(h)** Bar=4 cm. **(B)** The expression level of *CaTIFY10b* were analyzed in transgenic pepper plants under normal or cold stress. **(C-K)** The transcription level of *CaERD15*, *CaCBF1a*, *CaCBF1b*, *CaKIN*, *CaCOR47-like*, *CaSOD*, *CaPOD*, *CaCAT2* and *CaAPX1* were detected in transgenic pepper plants with TRV2:*CaTIFY7* under normal or cold stress. The gene expression levels were normalized to those of *CaUBI3*. All the values are the averages ± SD from three independent experiments. Different letters indicate significant differences between samples according to the Student-Newman-Keuls test (*P <*0.05).

**Figure 9:**
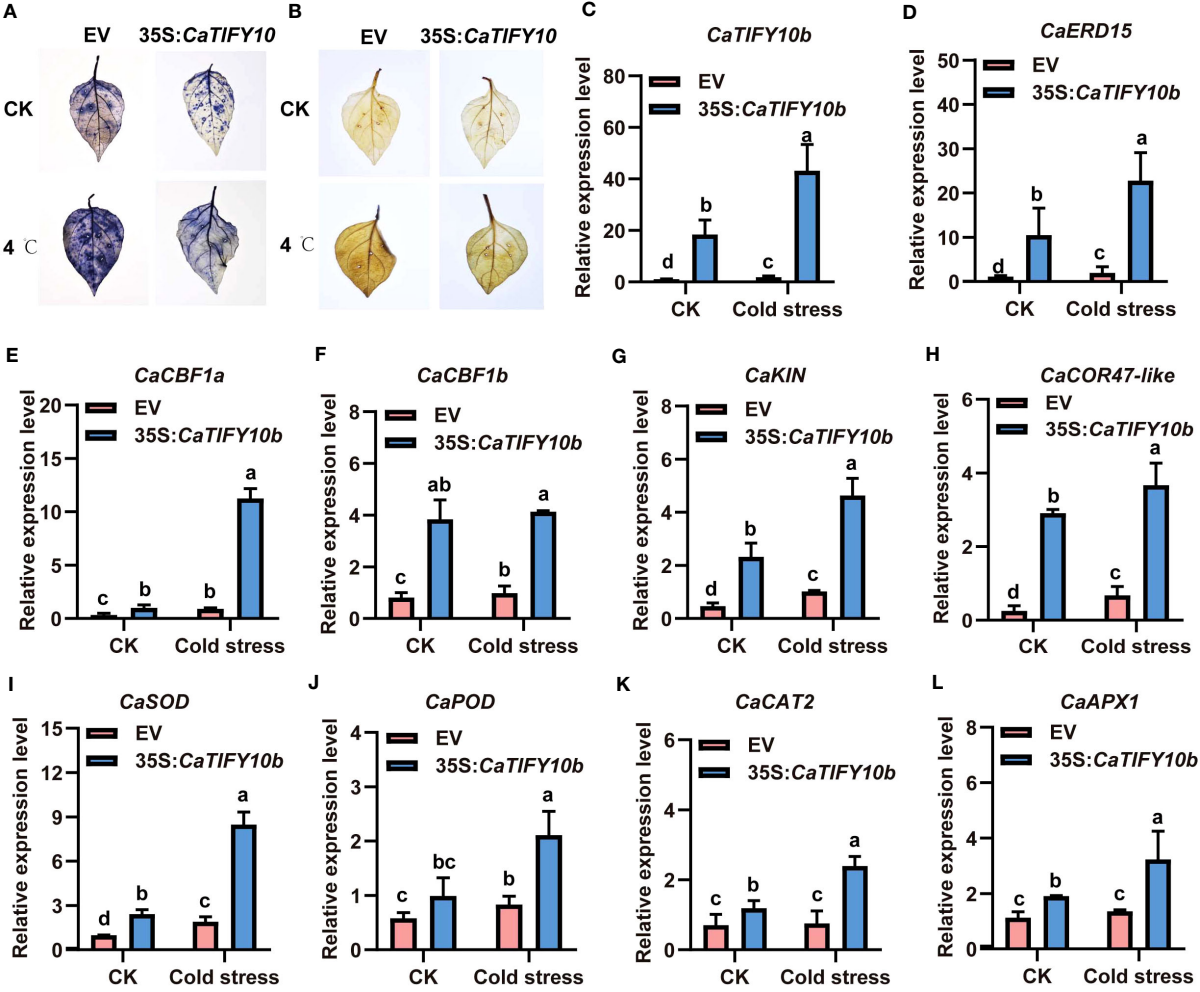
Overexpression of *CaTIFY10b* positively regulated cold stress response in pepper. **(A)** O_2_^-^ detecting with NBT under normal or cold stress. **(B)** H_2_O_2_ staining with DAB after cold stress. **(C)** The transcription level of *CaTIFY10b* were analyzed in transgenic pepper plants having 35S:*CaTIFY10b* under normal or cold stress. **(D-L)** The expression of cold stress and ROS-related genes were detected in transgenic pepper plants with 35S:*CaTIFY10b* after cold stress treatment. *CaUBI3* were used as the reference gene. All the values are the averages ± SD from three independent experiments. Different letters indicate significant differences between samples according to the Student-Newman-Keuls test (*P <*0.05).

To further confirm the function of *CaTIFY10b* in cold-stress response, transgenic pepper leaves containing the 35S:*CaTIFY10b* vector were generated to explore the role of *CaTIFY10b* in cold tolerance. After 24 h, the *CaTIFY10b*-silenced plants showed a more severe degree of withering than the control plants (**Figure 8A**). The RT-qPCR results showed that *CaTIFY10b* expression was successfully reduced in TRV2:*CaTIFY10b-*silenced plants (**Figure 8B**). Subsequently, the expression of cold-induced and ROS-related genes in *CaTIFY10b*-silenced pepper leaves was analyzed and the knockdown of *CaTIFY10b* significantly suppressed the expression of cold-induced and ROS-related genes under cold stress (**Figures 8C–K**). As expected, overexpression of *CaTIFY10b* also increased the reactive oxygen content in plant leaves and significantly increased the expression of these genes under cold stress in pepper (**Figures 9A–L**). These results showed that *CaTIFY10b* positively regulated cold tolerance in pepper by promoting the expression of stress-related genes.

### *CaTIFY7* interacted with CaTIFY10b in yeast

**Figure 10:**
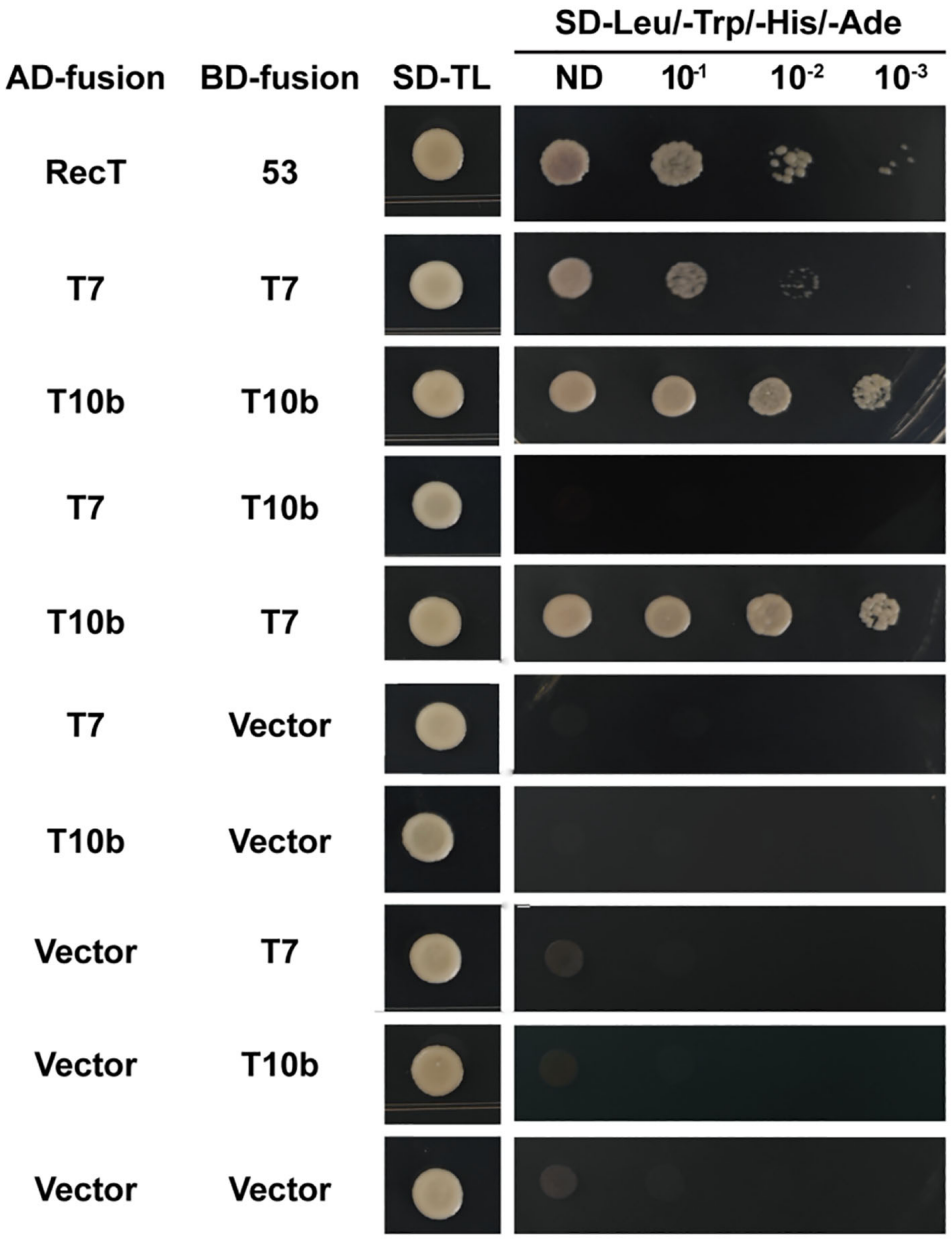
Y2H assay between CaTIFY7 and CaTIFY10b. CaTIFY7 interacts with itself or CaTIFY10b and CaTIFY10b interacts with itself or CaTIFY7 in the Y2H assay. Protein interactions were analyzed by the growth of yeast in the medium without leucine, tryptophan, histidine and adenine medium.

Because CaTIFY7 and CaTIFY10b are both involved in cold-stress reactions, it would be interesting to explore whether they can form heterodimers in response to cold-stress signals. The interaction network of the 16 CaTIFY proteins was predicted using the STRING database, and the results are shown in **Supplementary Figure 4**. Surprisingly, the results indicated that CaTIFY7 and CaTIFY10b may have a conserved interaction relationship in the model plant. To confirm this relationship, we performed a yeast two-hybrid analysis to verify their interaction. As shown in **Figure 10**, CaTIFY7 interacted with CaTIFY10b in the Y2H assay (**Figure 10**). In addition, CaTIFY7 and CaTIFY10b interacted with each other (**Figure 10**). These results showed that CaTIFY7 and CaTIFY10b may form heterodimers or homodimers to regulate the cold-stress response in pepper.

## Discussion

TIFY proteins are defined as TIFY transcription factors because of their specific binding to consensus sequences. TIFY proteins are important regulators of abiotic and biotic stress responses in plants. Several TIFY proteins have been identified in various plant species. However, the *TIFY* gene family in Solanaceae crops, including pepper, has not previously been identified or characterized. We identified sixteen *TIFY* genes in pepper and the *TIFY* gene family members all contained the conservative TIFY⁃motif and JAZ domains (**Figures 1**; **2**). The lack of a homologous gene for Arabidopsis *AtTIFY5* among the TIFY members in pepper suggests that this homologous gene may have undergone selective loss in pepper. All *CaTIFY* genes were distributed on chromosomes 1, 3, 4, 6, 7, 8, 9, and 12 and only *CaTIFY1b/c* formed a homologous gene cluster on chromosome 8 (**Figure 3**).

Many elements were related to hormones and stress in the *CaTIFYs* promoter of peppers, but there were significant differences among the different *CaTIFY* genes (**Figure 4**). Most members of the TIFY gene family in pepper had anaerobic induction elements, and more than half of the *TIFY* genes responded to ABA, MeJA, salicylic acid, and gibberellin. In addition, some genes contained different stress response elements, suggesting that *CaTIFYs* may be involved in the regulation of different hormone signal transduction pathways, thereby responding to environmental stress.

The TIFY family is widely distributed in plants and its members play important roles in regulating the development of stems, leaves, and flowers in plants (Zhu et al., 2011; Hakata et al., 2012; He et al., 2015; Baekelandt et al., 2018). To better understand the differential expression of *CaTIFYs* genes in peppers, we analyzed the gene expression of *CaTIFYs* in different tissues. RNA-seq data showed that the expression levels of the majority of pepper *TIFY* did not show tissue-specific expression patterns (**Supplementary Figures 2**, **3**). The expression levels of *CaTIFY7* were high in most tissues, suggesting that they have an important role in pepper development. Notably, *CaTIFY10* was most highly expressed in the roots, suggesting that it has a key role in the root development and growth of pepper.

Next, we analyzed the gene expression patterns of this family under different stress conditions (**Supplementary Figure 3**). Salt stress induced expression of *TIFY6b*, *TIFY8*, *TIFY10b*, and *TIFY10c* in roots, but repressed the transcription levels of *TIFY6b* and *TIFY4a* in leaves, suggesting that *TIFY6b* expression is regulated by different mechanisms in different tissues. The significant induction of *TIFY10b* and *TIFY10c* expression suggests that they are likely to participate in salt stress responses, which is consistent with the function of their homologous genes (*GmTIFY10e* and *GmTIFY10g*) in the positive regulation of salt tolerance in soybeans (Liu et al., 2022). Under drought stress, the induced expression of *TIFY10a*, *TIFY10b*, and *TIFY10c* in leaves and roots indicates that *TIFY10* might play an important role in the drought response of pepper. Based on the discovery that multiple members of the TIFY family enhance the drought resistance of different plants (Ye et al., 2009; Zhao et al., 2016; Zhang et al., 2023), it can be predicted that CaTIFY10a/b/c may also regulate drought resistance in pepper.

Among all *TIFY* genes, the responses of *TIFY6a*, *TIFY6b*, *TIFY7* and *TIFY10b* to high temperatures were the most significant (**Supplementary Figure 3**). The induced expression of *TIFY6a*, *TIFY6b*, and *TIFY10b*, and the inhibited expression of *TIFY7*, suggest that *TIFY6a*, *TIFY6b*, and *TIFY10b* might play a role in enhancing high-temperature resistance in pepper while the function of *TIFY10b* might be the opposite. However, there are almost no reports on the function of *TIFY* in responses of plants to high temperatures and this is a direction worth studying further. Similarly, the function of TIFY during the low-temperature response is unclear. In pepper, we found that several TIFY (*TIFY1a*, *TIFY4a*, *TIFY6a*, *TIFY6b*, *TIFY10a*, *TIFY10b*, and *TIFY10c)* genes responded to cold stress, suggesting that they may be important candidate genes for the cold response in pepper.

To cope with low-temperature stress, plants have evolved a “cold adaptation” mechanism. Many transcription factors play an important role in the regulation of cold-stress adaptation in plants (Chinnusamy et al., 2010; Ding et al., 2019). It has long been known that the *TIFY* gene responds to cold stress in rice, rape, tomato, bamboo, and grain sorghum (Ye et al., 2009; Huang et al., 2016; Chini et al., 2017; He et al., 2020; Du et al., 2022). However, there have been few reports on the function of *TIFY* in plant responses to low temperatures. Our results confirmed that pepper *CaTIFY7* and *CaTIFY10b* were positive regulators of plant responses under cold stress, where they regulated the expression levels of cold-induced and ROS-related genes (**Figures 6**–**9**). We also demonstrated that *CaTIFY7* and *CaTIFY10b* play important roles in pepper plants under salt stress.

To further analyze the regulatory mechanism of *CaTIFY7* and *CaTIFY10b* in terms of protein interactions, we analyzed the CaTIFY protein interaction network using the STRING database (**Supplementary Figure 4**). Many CaTIFYs, except for CaTIFY1b, CaTIFY1c, CaTIFY1a, and CaTIFY2, interacted with each other to form complexes, indicating that most CaTIFYs formed heterodimers that may affect protein function. Some members of the CaTIFY family formed a complex regulatory network with COI1 protein, MYC2 transcription factor, and Ninja and bHLH13 proteins. It’s known that COI1/JAZs/MYC2 acts as the core jasmonic acid-signaling module in the plant jasmonic acid signaling pathway (Chini et al., 2009; Chung and Howe, 2009), which means that these CaTIFYs have a conserved function in sensing JA signals and regulating JA levels in pepper. However, CaTIFY1a/b/c and CaTIFY2 are independent of this signaling pathway, suggesting that these genes may exert regulatory functions through unique mechanisms. Interestingly, CaTIFY7 interacted with CaTIFY10b, as demonstrated by the Y2H assay (**Figure 10**), suggesting that CaTIFY7 and CaTIFY10b act as heterodimers or homodimers to regulate the cold-stress response in pepper.

## Conclusions

We identified 16 *CaTIFY* genes in pepper, of which *CaTIFY7* and *CaTIFY10b* were significantly upregulated by cold stress. Silencing *CaTIFY7* and *CaTIFY10b* significantly repressed the cold tolerance of transgenic pepper plants and the expression of cold-induced and ROS-related genes. Conversely, overexpression of *CaTIFY7* and *CaTIFY10b* enhanced cold tolerance by promoting the expression of cold-induced and ROS-related genes. Further, we showed that CaTIFY7 and CaTIFY10b interacted with each other and that CaTIFY7 interacted with CaTIFY10b. This study provides a basis for further research on how TIFY family members regulate cold tolerance in plants.

## Data availability statement

The original contributions presented in the study are included in the article/**Supplementary Material**. Further inquiries can be directed to the corresponding authors.

## Author contributions

XW: Data curation, Formal Analysis, Investigation, Methodology, Software, Writing – original draft, Writing – review & editing. NL: Writing – original draft, Conceptualization, Data curation, Formal Analysis, Writing – review & editing. TZ: Writing – original draft, Investigation, Project administration. KX: Conceptualization, Data curation, Writing – original draft, Formal Analysis. SG: Conceptualization, Data curation, Project administration, Writing – original draft. YY: Conceptualization, Project administration, Resources, Writing – original draft. MY: Funding acquisition, Supervision, Validation, Writing – review & editing. FW: Funding acquisition, Supervision, Validation, Writing – review & editing.
