# Supplementary material for: Genome-wide analysis of the TIFY family and function of CaTIFY7 and CaTIFY10b under cold stress in pepper (Capsicum annuum L.)
Source: Front Plant Sci. 2023 Nov 22;14:1308721. doi: 10.3389/fpls.2023.1308721 (PMC10702603; doi:10.3389/fpls.2023.1308721)
Supplement: Supplementary file 1 [file DataSheet_1.doc]

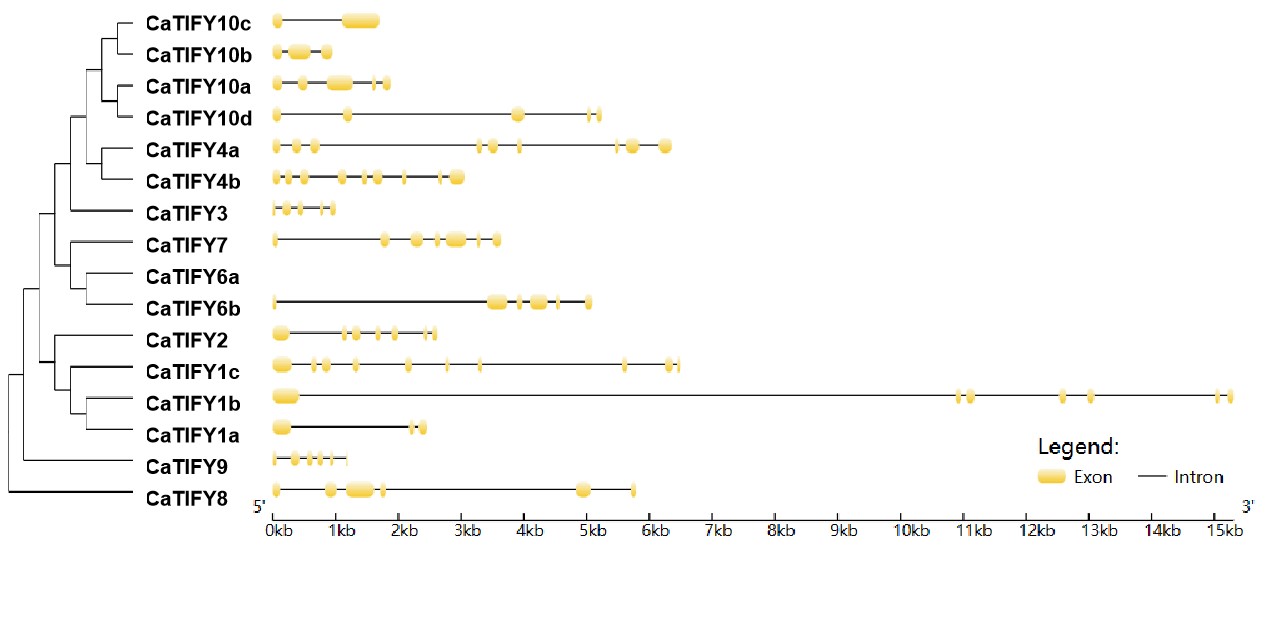


**FIGURE S1. Phylogenetic relationships and gene structure of *TIFY* genes in pepper.**

The phylogenetic tree and exon/intron distribution of the sixteen pepper *TIFY* genes were determined by the GSDS tool. The yellow box depicts the CDS and the solid black represents line the intron region.


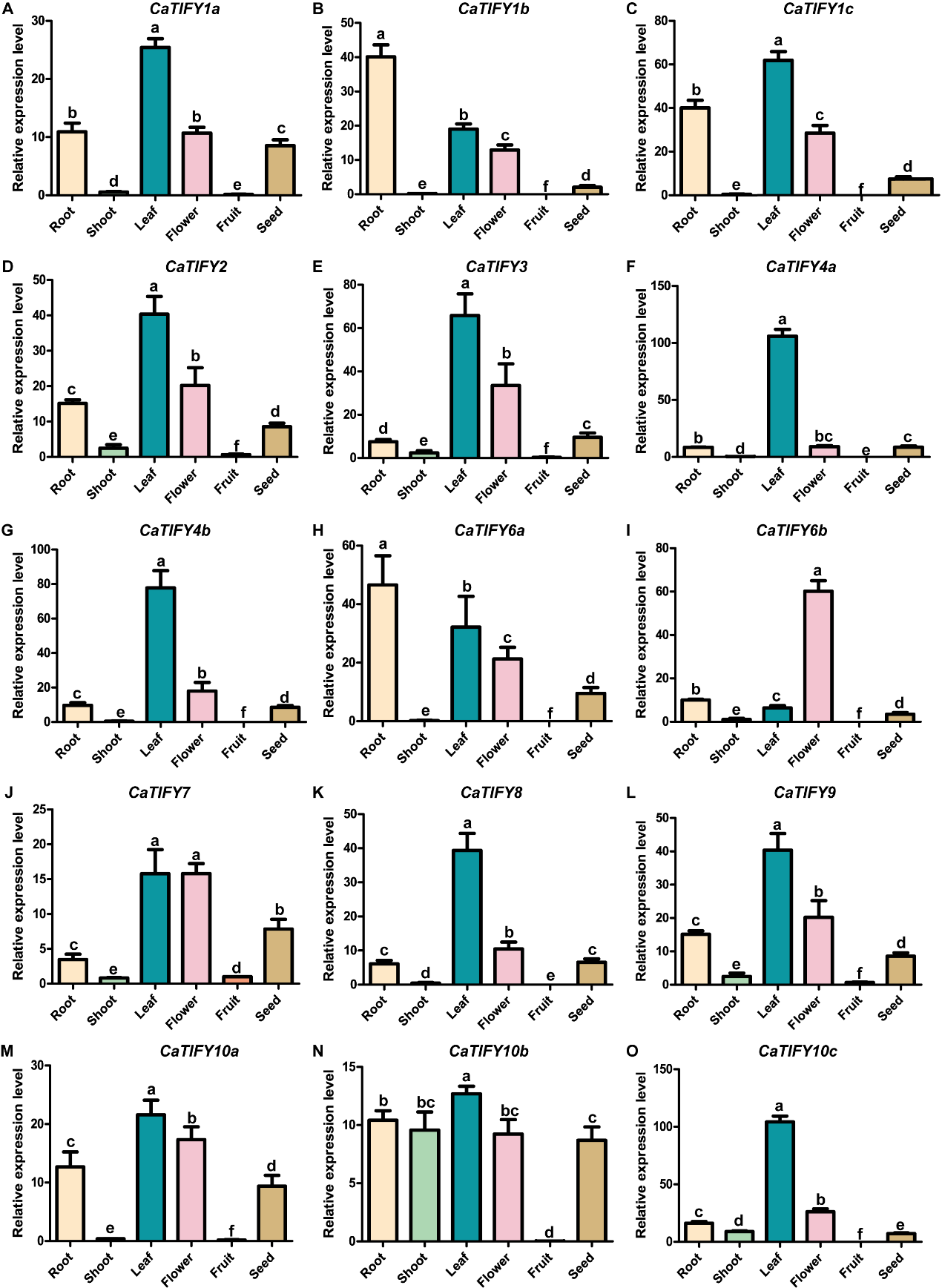

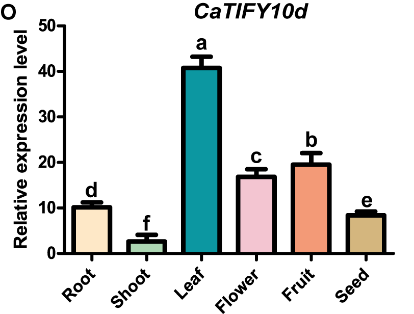


**FIGURE S2 Expression of *CaTIFY* genes in different tissues.** *CaUBI3* was used as an reference gene. The relative transcript level was determined and normalized using the reference level and averaged over the three technical replicates. Different letters indicate significant differences between samples according to the Student-Newman-Keuls test (*P* <0.05).

**
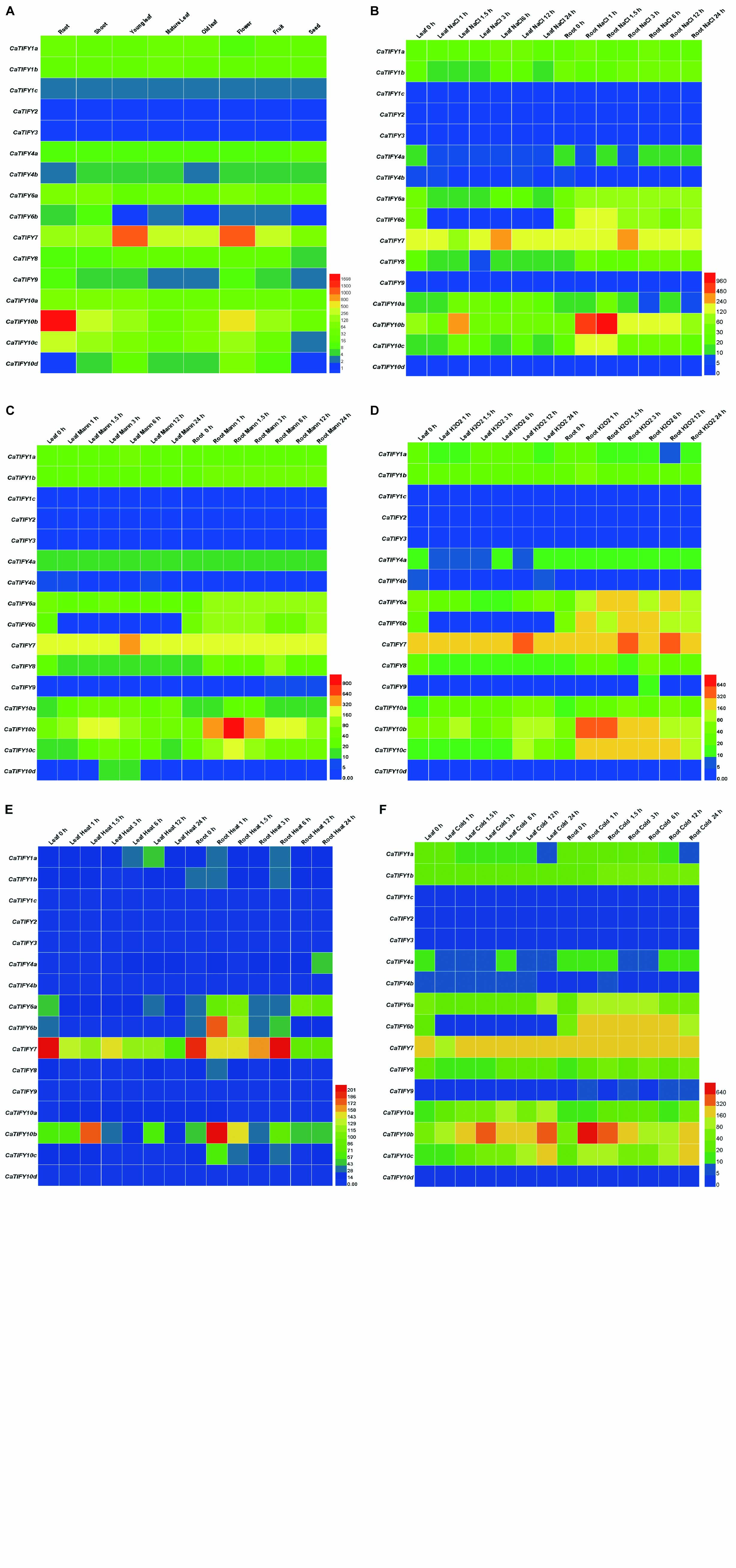
**

**FIGURE S3 Heatmap of *CaTIFY* genes in different tissues or under different abiotic stress**. The expression profles of 16 *CaTIFY* genes in root, shoot, young leaf, mature leaf, old leaf, flower, fruit and seed. The expression level of *CaTIFY* genes under different conditions including salt, H_2_O_2_, mannitol, heat and cold treatments. Heatmaps indicate the transcription levels of each *CaTIFY* gene (Log_2_ (FPKM values)) in each sample, as indicated by different color rectangles.

**
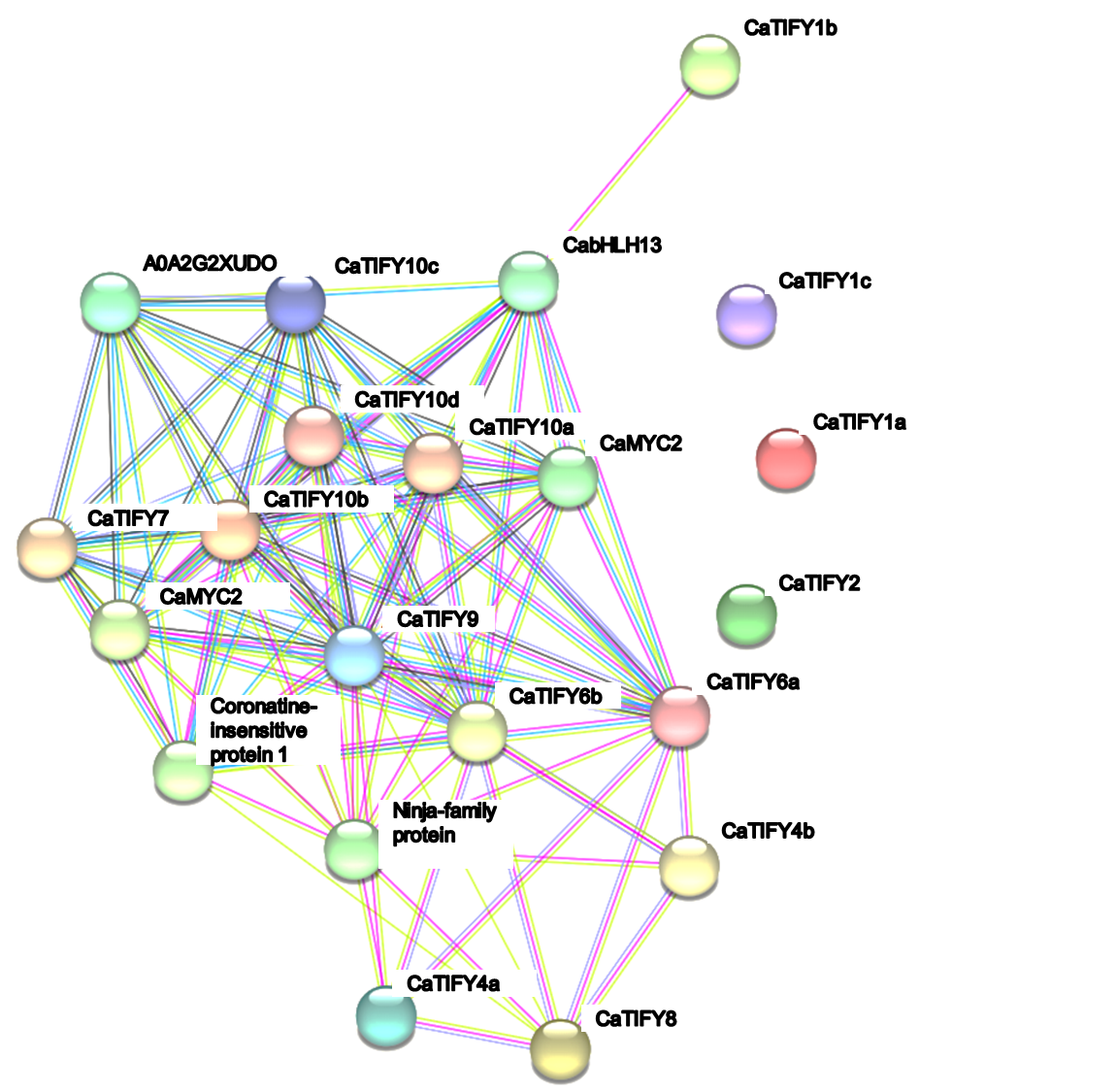
**

**FIGURE S4 Interaction network of CaTIFY proteins in pepper using the String database.**

**Table S1** List of primers used for quantitative real‐time PCR analysis and vector construction.

| *CaTIFY7*-TRV2-F | CTAGATGAGGAGAAGAGCCCGCTCCAAAGAAACTGGGTTTCGCTG |
| --- | --- |
| *CaTIFY7*-TRV2-R | TGTGCTCGACGACAAGACCCGAGGAGCCGTAACAGGAATTCCACC |
| *CaTIFY10b*-TRV2-F | CTAGATGAGGAGAAGAGCCCGGCTGGTCAGAGATCGCATTTCTC |
| *CaTIFY10b*-TRV2-R | TGTGCTCGACGACAAGACCCGGCGTTGTTACTAGCCATAAGCATG |
| *CaTIFY7*-pMV2-F | GAGGACACGCTCGACCTCGAGATGGAGAGGGACTTTATGGGAC |
| *CaTIFY7*-pMV2-R | AAGCAGGACTCTAGTGGTACCGGTCTCCTTACCGGCTAAAAGA |
| *CaTIFY10b*-pMV2-F | GAGGACACGCTCGACCTCGAGATGGCTTCATCAGAGATTGTGGA |
| *CaTIFY10b*-pMV2-R | AAGCAGGACTCTAGTGGTACCGTATTGCTCAGTTTTCACAGGAAAC |
| *CaTIFY7*-AD-F | TGGAGGCCAGTGAATTC ATGGAGAGGGACTTTATGGGAC |
| *CaTIFY7*-AD-R | TCGAGCTCGATGGATCCT GGTCTCCTTACCGGCTAAAAGA |
| *CaTIFY7*-BD-F | ATGGCCATGGAGGCCGAATTC ATGGAGAGGGACTTTATGGGAC |
| *CaTIFY7*-BD-R | CTAGTTATGCGGCCGCTGCAG GGTCTCCTTACCGGCTAAAAGA |
| *CaTIFY10b*-AD-F | TGGAGGCCAGTGAATTC ATGGCTTCATCAGAGATTGTGGA |
| *CaTIFY10b*-AD-R | TCGAGCTCGATGGATCCTGTATTGCTCAGTTTTCACAGGAAAC |
| *CaTIFY10b*-BD-F | ATGGCCATGGAGGCCGAATTC ATGGCTTCATCAGAGATTGTGGA |
| *CaTIFY10b*-BD-R | CTAGTTATGCGGCCGCTGCAGGTATTGCTCAGTTTTCACAGGAAAC |
| qPCR-*CaTIFY7-*F | CCAAAGGTTCTGCAGCACCTG |
| qPCR-*CaTIFY7*-R | GGTTTGGGTGCAGATGCCTG |
| qPCR-*CaTIFY10b*-F | GCGGAATCTACTGTTGATTTGG |
| qPCR-*CaTIFY10b*-R | GCTGTGTGTTTATGAGGGGTGC |
| qPCR-*CaCBF1a-*F | AATCTGCTGACAGTAGTTCTCC |
| qPCR-*CaCBF1a-*R | TCAACTTCCACATGATCTCCAA |
| qPCR-*CaCBF1b-*F | GGCTATAGCATTAAGAGGTCGT |
| qPCR-*CaCBF1b-*R | ATCTTTAGTGTCAGAGGAAGCC |
| qPCR-*CaKIN-*F | AAGCTCAGGAAAAGGGTAAC |
| qPCR-*CaKIN-*R | CAGTGGCGTTTTTAACTGCA |
| qPCR-*CaCOR47-like-*F | GCTAACGTGGAATCAACAGATC |
| qPCR-*CaCOR47-like-*R | ACTTCTTCCTCCTCATCACTTG |
| qPCR-*CaSOD-*F | TATGGAGCCTTAGAACCTGC |
| qPCR-*CaSOD-R* | CCATTGAACTTGATAGCACCT |
| qPCR-*CaPOD-*F | TCCTCCTCCTACTTCTAACC |
| qPCR-*CaPOD-R* | ACAGACCTCTTTTGCTCACT |
| qPCR-*CaCAT2-*F | GAAGCCAAATCCTAAGTCCC |
| qPCR-*CaCAT2-R* | CCAACTCGGATTGCCTCTT |
